# Association of Playing Cards or Mahjong with Cognitive Function in Chinese Older Adults

**DOI:** 10.3390/ijerph19159249

**Published:** 2022-07-28

**Authors:** Jin Wang, Nan Liu, Xiaoguang Zhao

**Affiliations:** 1 Faculty of Sport Science, Ningbo University, Ningbo 315211, China; wangjin@nbu.edu.cn (J.W.); 2111042019@nbu.edu.cn (N.L.); 2 Research Academy of Grand Health, Ningbo University, Ningbo 315211, China

**Keywords:** Chinese, cognitive decline, older adults, playing cards or mahjong, social entertainment

## Abstract

Cognitive decline in older adults is a major public health threat. This study aimed to explore the association of participation in cards or mahjong with cognitive function in older adults. A total of 7308 older adults were selected from the 2018 Chinese Longitudinal Healthy Longevity Survey. A modified Mini-Mental State Examination (MMSE) was used to assess cognitive function. The participants were classified according to the frequency of playing cards or mahjong into the “regularly” (R) group, “occasionally” (O) group, or “never” (N) group. The results showed that older persons in the R group and O group had better cognitive function than those in the N group. Specifically, significant differences were found in attention and calculation, language, and total MMSE score between the R group and the N group. However, significant differences were not observed for adults aged 60–69 years old. Regression analysis indicated that playing cards or mahjong, together with age, educational level, sex, marital status, and occupation before age 60 could explain the cognitive function. The findings suggest that there is an association between participation in cards or mahjong and cognitive function in the population of Chinese older adults, and that the frequency of participation plays an important role in the association.

## 1. Introduction

Population aging is one of the most critical issues that most countries around the world, including China, are facing. China officially became an aging society in 2000, when the proportion of the population aged over 60 years reached 10% [1]. The latest national population census data in China show that the share of the population aged over 60 and 65 years grew from 13.26% in 2010 to 18.70% in 2020 and from 8.9% to 13.5% in the past ten years, respectively [2]. By 2050, it is estimated that China will have 400 million people aged above 65 years, accounting for approximately 25% of the total population, and that the population aged over 80 years and older will reach 150 million [3,4]. In light of this, more attention should be given to the health problems of older adults caused by the improvement in life expectancy. 

Cognitive decline is a major public health threat to older adults. In China, a previous cohort study showed that the prevalence of subjective cognitive decline was 18.8% for people aged 60 to 80 years [5]. Moreover, a nationwide cross-sectional study indicated that the prevalence of mild cognitive impairment was 15.5% (nearly 38.8 million adults) for persons aged 60 years and older [6]. Cognitive decline is closely associated with normal aging and is a leading cause of Alzheimer’s disease and dementia that result in a decline in daily functioning [7]. Multiple studies have revealed that cognitive decline has a wide variety of adverse effects on mental and physical health [8,9,10], which places an enormous burden on families and the medical care system [11,12]. 

There are several non-modifiable risk factors and modifiable risk factors that affect cognitive function. Non-modifiable risk factors include gender, age, ethnicity, heredity, and chronic diseases [13,14,15], whereas modifiable risk factors include physical activity, nutritional supplements, smoking, and alcohol abuse [16,17,18]. Additionally, recent studies have demonstrated that performing intellectual activities or games related to mental workload has a beneficial effect on cognitive function in older persons [19,20].

Playing cards or mahjong is a highly intellectual and mental demanding task [21]. In China, it is a highly popular social entertainment program for older adults. A primary reason for the popularity of the game is that it has a gambling-like win-or-lose property; another reason may be that there are various playing patterns and strategies for the game [19]. In order to win or play well in the game, players need to coordinate and cooperate a variety of abilities such as attention, observation, alertness, memory, recall, calculation, language, and communication. These abilities are repeatedly used in the game, which may improve and maintain the cognitive function of older people. Chu-Man et al. [22] demonstrated that engaging in cards or mahjong is an effective way to improve attention, short-term memory, and logical reasoning capability in both middle-aged and older adults. In addition, participation in cards or mahjong has been reported to promote psychological well-being in middle- and old-aged adults by providing social communication and social interaction [23,24]. Zhang and colleagues [19] suggested that interpersonal interactions, and enriched emotional and environmental stimulation during cards or mahjong activity, may have an important impact on the reactivation of neural circuits in the brain.

In order to prevent or delay cognitive decline, it is necessary to determine which factors influence cognitive function. Although many studies have demonstrated that regular exercise may be an effective strategy to prevent or improve cognitive decline [25,26,27], studies concerning the relationship between entertainment activities and cognitive function are relatively scarce. Therefore, this study aimed to determine the association of playing cards or mahjong with cognitive function in older adults. The study hypothesized that older adults who played cards or mahjong have a higher level of cognitive function than those who do not participate in such games.

## 2. Materials and Methods

### 2.1. Study Population and Data Source

**Figure 1:**
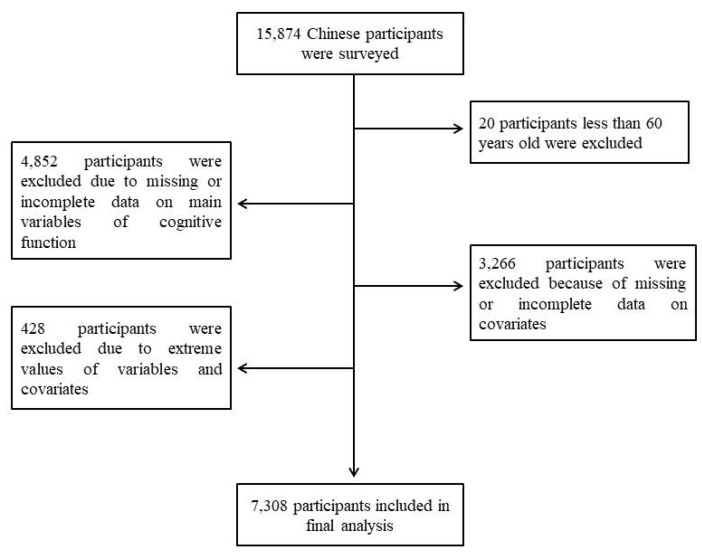
Flow diagram of participants’ study exclusion and inclusion.

This is a retrospective cross-sectional study based on the 2018 national wave of the Chinese Longitudinal Healthy Longevity Survey (CLHLS 2018). The CLHLS conducted a face-to-face interview in 22 provinces in China using internationally compatible questionnaires on demographics, family, disability, health, and behavioral, and socioeconomic risk factors. Based on the objective of our study, we created the inclusion criteria for the study participants as follows: (a) individuals aged 60 years and older, (b) complete information on cognitive function, (c) complete data on covariates including sex, age, and educational level, and (d) the values of variables and covariates were within the normal range. The exclusion criteria in our study were as follows: (a) adults who were diagnosed with dementia and Alzheimer’s disease, (b) persons who had visual, auditory, or linguistic impairment, and (c) adults who stayed in bed for long periods. In the CLHLS 2018, there were a total of 15,874 Chinese people included in the study, 20 of whom less than 60 years old had to be excluded, 4852 of whom were excluded due to missing or incomplete data on main variables of cognitive function, 3266 of whom were excluded because of missing or incomplete data on covariates, and 428 of whom were excluded due to extreme values of variables and covariates. Consequently, the final sample consisted of 7308 participants in the study. Figure 1 shows a flow chart of participants’ study exclusion and inclusion. All the participants were given a clear explanation of the procedures and purposes of this study, and a written informed consent form was obtained from them. The CLHLS project has been reviewed and approved by the Biomedical Ethics Committee of Peking University (approval number: IRB00001052-13074).

### 2.2. Playing Cards or Mahjong

The frequency of playing cards or mahjong was recorded by asking the participants to report how often they participated in the games in their daily life. The types or kinds of cards and mahjong the participants played was not limited in this study. Detailed information on the different types of cards and mahjong and how to play the games in China can be found elsewhere (https://www.pagat.com/national/china.html, accessed on 17 June 2022; https://www.chinaeducationaltours.com/guide/culture-mahjong.htm, accessed on 17 June 2022; https://www.wikihow.com/Play-Mahjong, accessed on 17 June 2022). Based on the frequency of participation in cards or mahjong, the participants in our study were classified into the “regularly” (R) group, “occasionally” (O) group, or “never” (N) group. In the study, the R group includes participants who, at the time of asking, played cards or mahjong almost every day or not daily but at least once a week. The O group involves participants who, at the time of asking, played cards or mahjong not weekly but at least once a month or not monthly but sometimes. The N group includes participants who, at the time of asking, never played cards or mahjong.

### 2.3. Cognitive Function

A Chinese version of the modified Mini-Mental State Examination (MMSE) was employed to assess the cognitive function of older adults in this study. The MMSE was modified by the CLHLS study group to facilitate older adults’ better understanding and response. It has been widely used in previous studies and has been proven to have good validity and reliability [28,29,30]. The modified MMSE includes 12 items in 5 dimensions such as orientation (score ranges, 0–5), registration (score ranges, 0–3), attention and calculation (score ranges, 0–5), recall (score ranges, 0–3) and language (score ranges, 0–7). The total score of the MMSE ranges between 0 and 23; lower scores represent poorer cognitive function, and vice versa. In this cross-sectional study, the Cronbach’s alpha for the modified MMSE was 0.822.

### 2.4. Covariates

In this study, covariates were composed of sex, age, anthropometry, marital status, co-residence, occupation before age 60, current smoker, current drinker, exercise habits, educational level, and annual income. Anthropometry included body weight, height, and body mass index (BMI). Marital status was classified into married and living with spouse, separated, divorced, widowed, and never married. Co-residence was divided into with household member(s), alone, and in an institution. Occupation before age 60 included professional and technical personnel, governmental, institutional or managerial personnel, commercial service or industrial worker, self-employed, and so on. Participants were identified as current smokers or current drinkers if they smoke or drank at the time of asking. Participants were identified as having exercise habits if they exercised regularly at that time. Educational level and annual income, as continuous variables, were assessed as years of schooling and total income of household last year, respectively.

### 2.5. Statistical Analysis

We obtained the data from the CLHLS 2018 cross-sectional dataset in SPSS format. First, descriptive statistics was used to describe the characteristics of involved participants. The data were expressed as numbers and percentages (%) or mean ± standard deviation (SD). Second, we used one-way ANOVA followed by Bonferroni post hoc multiple comparisons to determine the relationship between playing cards or mahjong and MMSE items in older adults stratified by age. Finally, multiple regression analysis was employed for exploring which components were associated with cognitive function. The total MMSE score was set as the dependent variable, and the playing cards or mahjong and other covariates with *p* values less than 0.10 in the univariate analysis similar to previous studies [31,32], were set as the independent variables. Stepwise multiple linear regression analysis was employed to obtain variables that were retained in the final model, and regression coefficient (B), standard error (SE), T value, and R^2^ were used to present results. Data processing and analysis were performed using IBM SPSS software version 25.0 (SPSS Inc., Chicago, IL, USA), and a level of significance lower than 0.05 was deemed statistically significant in the statistical analysis.

## 3. Results

The demographic characteristics and MMSE scores of 7308 adults aged 60 years or older are shown in Table 1. There were 4039 (55.27%) men and 3269 (44.73%) women in the sample, with a mean BMI of 23.09 ± 3.90 kg/m^2^. More than 56% of older persons were married and living with spouse and more than 81% of them lived with household member(s). Nearly half of the participants engaged in agriculture, forestry, animal husbandry or fishery before the age of 60. The mean educational level was 5.06 ± 4.52 years and the annual income was RMB 47,619.80 ± 37,601.59. The proportions of participants who regularly, occasionally, and never played cards or mahjong were 16.67%, 8.35%, and 74.99%, respectively. The mean MMSE score of 7308 participants was 21.62 ± 2.35. 

Table 2 presents the relationship between engaging in cards or mahjong and MMSE items in all participants. There were statistically significant differences in all MMSE items including orientation, registration, attention and calculation, recall, language, and total MMSE score (all *p* < 0.01) among the three different groups of playing cards or mahjong. A post hoc analysis showed that both the R group and O group had a higher score in all MMSE items (all *p* < 0.05) compared to the N group. However, no statistically significant differences were found between the R group and O group for any MMSE items.

The relationships between playing cards or mahjong and MMSE items stratified by age are described in Table 3. For participants aged 60–69 years old, there was no significant difference in any MMSE items among the three different groups of playing cards or mahjong. For participants aged 70–79 years old, statistically significant differences were observed in attention and calculation, language, and total MMSE score (*p* < 0.05) among the three different groups. A post hoc analysis indicated that compared to the N group, both the R group and O group had greater scores for attention and calculation, language, and total MMSE score (*p* < 0.05). For participants aged 80–89 years old, the N group had a lower score in registration, attention and calculation, language, and total MMSE score than the R group (*p* < 0.05), and had a lower score in attention and calculation, and total MMSE score than the O group (*p* < 0.05). For participants aged 90 years and older, the N group had a lower score in attention and calculation, language, and total MMSE score than the R group (*p* < 0.05). However, no significant difference was found in any MMSE items between the N group and the O group.

Table 4 displays the results of stepwise regression analysis between MMSE score and playing cards or mahjong and other covariates. It was observed that age (B = −0.07, *p* < 0.001), educational level (B = 0.08, *p* < 0.001), sex (B = −0.28, *p* < 0.001), playing cards or mahjong (B = −0.08, *p* < 0.001), marital status (B = −0.06, *p* = 0.012), and occupation before age 60 (B = −0.05, *p* = 0.020) are the main components for explaining the MMSE score. The mean explanatory power of MMSE score composed of age, educational level, sex, playing cards or mahjong, marital status, and occupation before age 60 was 16% (R^2^).

## 4. Discussion

The prevalence of cognitive decline related to Alzheimer’s disease and dementia is increasing with the extension of life expectancy. The purpose of this study was to explore the association of playing cards or mahjong with cognitive function in older adults. The results showed that older people who regularly and occasionally played cards or mahjong had better cognitive function compared to those who never played. Specifically, significant differences were found in attention and calculation, language, and total MMSE score between those who regularly and never played cards or mahjong. However, significant differences were not observed for adults aged 60–69 years old. Stepwise regression analysis showed that playing cards or mahjong, together with age, educational level, sex, marital status, and occupation before age 60 can explain the cognitive function in older adults. The findings are consistent with the hypothesis that older adults who played cards or mahjong have a higher level of cognitive function than those without participation in the games.

Playing cards or mahjong may sustain and improve the cognitive function of older people. It was found in this study that older people who regularly or occasionally played cards or mahjong performed better in orientation, registration, attention and calculation, recall, and language compared to those who never played. This finding is consistent with the results from previous studies. A cross-sectional study showed that participation in cards or reading books have a positive impact on cognitive functioning in adults aged 50 years or older [33]. Furthermore, a randomized controlled trial demonstrated that playing mahjong for 12 weeks can enhance executive function in older persons with mild cognitive impairment [19]. The main reason for these results is that playing cards or mahjong is a game that requires intellectual and mental activities. Specifically, players need to memorize, calculate, recall, pay attention and communicate during play in order to win or play well [22,34].

The relationships between playing cards or mahjong and cognitive function were varied for different age groups. In the present study, significant differences in MMSE scores were observed between the R group and the N group for people aged 70 years and older, but not found for people aged 60–69 years old. One plausible explanation for this result is that there were fewer people aged 60–69 years old with cognitive impairments. Consistent with this viewpoint, previous studies have noted that the prevalence of cognitive impairment in men aged 60–64 and 65–69 was 0.8% and 2.7%, and in women was 0% and 0.1%, respectively, which were far lower than that in adults aged 70 years and older [35]. 

In the current study, it was found that older people who regularly and occasionally played cards or mahjong had a higher MMSE score compared to those who never played, but no differences in MMSE scores were found between older adults who regularly and occasionally played cards or mahjong. This is similar to the results of previous studies. Zhou and colleagues [36] reported that a higher frequency of social participation (e.g., mahjong, cards, chess, or other activities) was related to a better level of cognitive function in middle- and old-aged adults. Mao et al. [37] noted that more frequently engaging in cards or mahjong, watching TV, and reading newspapers or books may lower the risk of cognitive impairment in adults aged 80 years or older. To summarize, these results indicate that the frequency of playing cards or mahjong has a key role in the association between playing cards or mahjong and cognitive function.

There are several factors that affect cognitive function. The factors can be divided into two categories, non-modifiable risk factors such as gender, age, ethnicity, heredity, and chronic diseases [13,14,15] and modifiable risk factors including physical activity, nutritional supplements, smoking, and alcohol abuse [16,17,18]. The results of the stepwise regression analysis in this study suggested that age, educational level, sex, playing cards or mahjong, marital status, and occupation before age 60 are the main components that explain the MMSE score. It is apparent that only playing cards or mahjong is the modifiable risk factor. Therefore, it is reasonable to assume that frequently engaging in cards or mahjong may prevent or delay cognitive decline in older people.

The detailed mechanism behind the association between playing cards or mahjong and cognitive function in older adults is unclear. However, it is possible to present several explanations for the association. One plausible explanation is based on the “use it or lose it” theory. The theory suggests that participation in cards or mahjong may enhance one’s intellectual and mental activities that are beneficial to better brain function or cognitive function [38]. Moreover, in addition to enriched emotional and environmental stimulation, playing cards or mahjong requires a certain amount of physical activity in upper extremities, which may increase cerebral blood flow, stimulate the central nervous system excitement, improve brain tissue metabolism, and promote the establishment of brain neural network, therefore resulting in adaptive changes in brain structure and function [23,39,40]. Additionally, participation in cards or mahjong also plays an important role in increasing interpersonal communication and social support, reducing psychological stress, thereby mitigating stress-related neuronal changes and improving cognitive function [41,42].

### Strengths and Limitations

The current study has several advantages. First, we determined the relationship between playing cards or mahjong and MMSE items in older adults stratified by different age groups. Second, a variety of covariates were controlled when we explored the association of playing cards or mahjong with cognitive function in older adults. Third, according to the frequency, playing cards or mahjong was divided into three categories rather than a dichotomy of “yes or no”, so different frequencies on the association between playing cards or mahjong and cognitive function could be investigated in this study.

There are several limitations to this retrospective cross-sectional study. First of all, a modified MMSE was used to measure the cognitive function of older adults in this study. Although the modified MMSE has been previously confirmed to have good validity and reliability [28,29,30], some differences exist between the modified MMSE and the original MMSE. Moreover, several covariates such as taking medications or not, nutritional status, diseases, and disorders, were not included during the statistical analysis, which may result in biases in the interpretation of results. Finally, it was a retrospective cross-sectional study based on the CLHLS 2018, which cannot draw the cause–effect association between playing cards or mahjong and cognitive function in older adults. A longitudinal study is warranted to verify the findings.

## 5. Conclusions

The aim of the study was to explore the association of playing cards or mahjong with cognitive function in older adults. The current findings suggest that there is an association between playing cards or mahjong and cognitive function in the population of Chinese older adults, and that the frequency of playing cards or mahjong has an important role in the association. These findings have implications for healthcare policy and support encouraging a higher frequency of playing cards or mahjong to protect against cognitive decline in Chinese older adults.

## Figures and Tables

**Table 1:** The demographic characteristics and MMSE scores of involved participants (*n* = 7308).

Variables	Category	Numbers	Percentage (%) or Mean ± SD
Sex			
	Men	4039	55.27
	Women	3269	44.73
Age			
	60–69, years	1273	17.40
	70–79, years	2697	36.90
	80–89, years	1971	27.00
	≥90, years	1367	18.70
Anthropometry			
	Height, cm	7308	158.12 ± 12.86
	Weight, kg	7308	58.26 ± 12.86
	BMI, kg/m^2^	7308	23.09 ± 3.90
Marital status			
	Married and living with spouse	4124	56.43
	Separated	168	2.30
	Divorced	35	0.48
	Widowed	2914	39.87
	Never married	67	0.92
Co-residence			
	With household member(s)	5973	81.73
	Alone	1123	15.37
	In an institution	212	2.90
Occupation before age 60			
	Professional and technical personnel	831	11.37
	Governmental, institutional, or managerial personnel	501	6.86
	Commercial, service, or industrial worker	1422	19.46
	Self-employed	156	2.13
	Agriculture, forestry, animal husbandry, or fishery worker	3627	49.63
	Houseworker	442	6.05
	Military personnel	82	1.12
	Never worked	79	1.08
	Others	168	2.30
Current smokers			
	Yes	1403	19.20
	No	5905	80.80
Current drinkers			
	Yes	1349	18.46
	No	5959	81.54
Exercise habits			
	Yes	3075	42.08
	No	4233	57.92
Educational level		7308	5.06 ± 4.52
Annual income		7308	47,619.80 ± 37,601.59
Playing cards or mahjong			
	Regularly	1218	16.67
	Occasionally	610	8.35
	Never	5480	74.99
Modified MMSE score			
	Orientation	7308	4.90 ± 0.43
	Registration	7308	2.90 ± 0.44
	Attention and calculation	7308	4.61 ± 1.06
	Recall	7308	2.63 ± 0.81
	Language	7308	6.57 ± 0.69
	Total MMSE score	7308	21.62 ± 2.35

Note: BMI, body mass index; MMSE, Mini-Mental State Examination.

**Table 2:** The relationship between playing cards or mahjong and MMSE items in 7308 Chinese older adults.

Variables	Playing Cards or Mahjong			F Value	*p* Value ^a^
	R Group (*n* = 1218)	O Group (*n* = 610)	N Group (*n* = 5480)		
Orientation	4.94 ± 0.28 *	4.95 ± 0.28 *	4.89 ± 0.47	14.01	<0.001
Registration	2.93 ± 0.36 *	2.93 ± 0.36 *	2.89 ± 0.46	7.22	0.001
Attention and calculation	4.81 ± 0.68 *	4.83 ± 0.66 *	4.54 ± 1.15	48.58	<0.001
Recall	2.72 ± 0.70 *	2.70 ± 0.70 *	2.60 ± 0.84	12.27	<0.001
Language	6.68 ± 0.53 *	6.71 ± 0.55 *	6.53 ± 0.73	34.62	<0.001
Total MMSE score	22.09 ± 1.59 *	22.12 ± 1.61 *	21.46 ± 2.53	52.81	<0.001

Note: R group, “regularly” group; O group, “occasionally” group; N group, “never” group. ^a^ From ANCOVA for comparing a difference among the 3 groups adjusted by sex, age, educational level, and marital status. * *p* < 0.05 compared to the N group using Bonferroni’s post hoc multiple comparisons.

**Table 3:** The relationship between playing cards or mahjong and MMSE items in older adults stratified by age.

Variables	Playing Cards or Mahjong			F Value	*p* Value ^a^
	R Group	O Group	N Group		
**Participants aged 60–69 years old**	*n* = 266	*n* = 170	*n* = 837		
Orientation	4.99 ± 0.11	4.98 ± 0.15	4.96 ± 0.24	1.93	0.145
Registration	2.96 ± 0.33	2.94 ± 0.36	2.96 ± 0.27	0.67	0.512
Attention and calculation	4.93 ± 0.41	4.89 ± 0.59	4.83 ± 0.73	2.55	0.078
Recall	2.80 ± 0.61	2.82 ± 0.54	2.82 ± 0.56	0.07	0.937
Language	6.75 ± 0.45	6.82 ± 0.40	6.75 ± 0.49	1.44	0.237
Total MMSE score	22.43 ± 1.10	22.45 ± 1.04	22.33 ± 1.44	1.01	0.363
**Participants aged 70–79 years old**	*n* = 534	*n* = 252	*n* = 1911		
Orientation	4.97 ± 0.20	4.96 ± 0.21	4.94 ± 0.30	1.97	0.140
Registration	2.93 ± 0.34	2.96 ± 0.30	2.95 ± 0.30	0.80	0.451
Attention and calculation	4.87 ± 0.53 *	4.89 ± 0.51 *	4.73 ± 0.86	9.70	<0.001
Recall	2.77 ± 0.61	2.73 ± 0.65	2.72 ± 0.70	1.01	0.365
Language	6.73 ± 0.48 *	6.78 ± 0.50 *	6.65 ± 0.60	7.94	<0.001
Total MMSE score	22.27 ± 1.28 *	22.33 ± 1.40 *	21.99 ± 1.81	8.76	<0.001
**Participants aged 80–89 years old**	*n* = 260	*n* = 127	*n* = 1584		
Orientation	4.95 ± 0.26	4.98 ± 0.12	4.90 ± 0.42	4.08	0.017
Registration	2.95 ± 0.27 *	2.94 ± 0.26	2.87 ± 0.49	4.65	0.010
Attention and calculation	4.81 ± 0.63 *	4.80 ± 0.62 *	4.45 ± 1.23	15.33	<0.001
Recall	2.69 ± 0.72	2.57 ± 0.76	2.58 ± 0.85	2.06	0.128
Language	6.61 ± 0.54 *	6.57 ± 0.60	6.50 ± 0.67	4.07	0.017
Total MMSE score	22.01 ± 1.43 *	21.88 ± 1.42 *	21.30 ± 2.36	14.55	<0.001
**Participants aged ≥90 years old**	*n* = 158	*n* = 61	*n* = 1148		
Orientation	4.82 ± 0.57	4.75 ± 0.67	4.73 ± 0.77	1.16	0.314
Registration	2.84 ± 0.52	2.79 ± 0.64	2.76 ± 0.67	1.17	0.312
Attention and calculation	4.46 ± 1.24 *	4.51 ± 1.18	4.14 ± 1.54	4.52	0.011
Recall	2.47 ± 1.00	2.43 ± 1.02	2.29 ± 1.09	2.20	0.111
Language	6.47 ± 0.72 *	6.41 ± 0.80	6.23 ± 0.99	5.16	0.006
Total MMSE score	21.06 ± 2.70 *	20.89 ± 2.93	20.14 ± 3.63	5.63	0.004

Note: R group, “regularly” group; O group, “occasionally” group; N group, “never” group. ^a^ From ANCOVA for comparing a difference among the 3 groups adjusted by sex, age, educational level, and marital status. * *p* < 0.05 compared to the N group using Bonferroni’s post hoc multiple comparisons.

**Table 4:** Stepwise regression analysis between total MMSE scores and playing cards or mahjong and other covariates (*n* = 7308).

	B	SE	T Value	*p* Value	R^2^
(Constant)	27.52	0.31	88.74	<0.001	0.16
Age	−0.07	0.01	−19.87	<0.001	
Educational level	0.08	0.01	9.77	<0.001	
Sex	−0.28	0.06	−4.68	<0.001	
Playing cards or mahjong	−0.08	0.02	−3.92	<0.001	
Marital status	−0.06	0.02	−2.52	0.012	
Occupation before age 60	−0.05	0.02	−2.32	0.020	

## Data Availability

Publicly available datasets were analyzed in this study. These data can be found here: [https://opendata.pku.edu.cn/dataverse/CHADS, accessed on 11 November 2021].
